# Ischemic priapism as a model of exhausted metabolism

**DOI:** 10.14814/phy2.13999

**Published:** 2019-03-27

**Authors:** Sanne Vreugdenhil, Pedro J. Freire Jorge, Mels F. van Driel, Maarten W. Nijsten

**Affiliations:** ^1^ Department of Urology University Medical Center Groningen University of Groningen Groningen Netherlands; ^2^ Department of Critical Care University Medical Center Groningen University of Groningen Groningen Netherlands

**Keywords:** Corpus cavernosum, glycopenia, lactic acidosis, priapism, respiratory acidosis

## Abstract

In vivo metabolic studies typically concern complex open systems. However, a closed system allows better assessment of the metabolic limits. Ischemic priapism (IP) constitutes a special model of the compartment syndrome that allows direct sampling from a relatively large blood compartment formed by the corpora cavernosa (CC). The purpose of our study was to measure metabolic changes and the accumulation of end products within the CC during IP. Blood gas and biochemical analyses of aspirates of the CC were analyzed over an 8‐year period. Mean ± SD pH, pCO_2_, pO_2_, O_2_‐saturation, lactate, and glucose of the aspirated blood were determined with a point‐of‐care analyzer. Forty‐seven initial samples from 21 patients had a pH of 6.91 ± 0.16, pCO_2_ of 15.3 ± 4.4 kPa, pO_2_ of 2.4 ± 2.0 kPa, and an O_2_‐saturation of 19 ± 24% indicating severe hypoxia with severe combined respiratory and metabolic acidosis. Glucose and lactate levels were 1.1 ± 1.5 and 14.6 ± 4.8 mmol/L, respectively. pH and pCO_2_ were inversely correlated (*R* ^2^ = 0.86; *P* < 0.001), glucose and O_2_‐saturation were positively correlated (*R* ^2^ = 0.83; *P* < 0.001), and glucose and lactate were inversely correlated (*R* ^2^ = 0.72; *P* < 0.001). The positive correlation of CO_2_ and lactate (*R* ^2^ = 0.69; *P* < 0.001) was similar to that observed in vitro*,* when blood was titrated with lactic acid. The observed combined acidosis underscores that IP behaves as a closed system where severe hypoxia and glycopenia coexist, indicating that virtually all energy reserves have been consumed.

## Introduction

Ischemic priapism (IP) is a persistent and usually painful erection lasting >4 h marked by maximal rigidity of the corpora cavernosa (CC) with little or no arterial inflow and no venous outflow. IP is considered to be a (penile) compartment syndrome characterized by an elevated intracorporal pressure that exceeds the arterial pressure (Lue 2016). The endothelium and the smooth muscle cells of the trabecular network in the CC are exposed to stagnant blood which becomes progressively more hypoxic and acidotic (Salonia et al. 2014; Lue 2016). Inadequate or delayed treatment of IP results in irreversible erectile dysfunction due to necrosis of these smooth muscles cells (Spycher and Hauri 1986).

IP is a unique model that offers the possibility to analyze regional metabolic changes in a closed system with accurate standard analytical techniques. We assessed a number of relevant metabolic parameters in a comprehensive manner which, to our knowledge, has never been performed before in patients with IP. By relating acid‐base parameters, oxygenation, glucose, and lactate, we tried to assess whether IP indeed behaves as a closed system and if metabolic limits were reached within it.

## Materials and Methods

We retrospectively analyzed patients who were presented with IP at the emergency department of the University Medical Center Groningen (UMCG) between January 2007 and July 2015. We collected anonymized data from medical records in the electronic hospital information system files including age, estimated duration, and cause of IP.

In a lithium heparin anticoagulated sample, 1 mL of blood was aspirated from the intercommunicating CC using a syringe with a disposable Gauche 30, 19 mm needle (Neofly^®^, yellow). In some cases, a local penis block was first provided at the dorsal base of the penis above the pubic bone (10 mL lidocaine 1%). Biochemical analyses included blood gasses, glucose, and lactate, performed with a point‐of‐care Radiometer 700 and 800 series analyzers (Radiometer Copenhagen), present at our emergency department for immediate measurements with a delay of only a few minutes, minimizing preanalytical errors.

**Figure 1:**
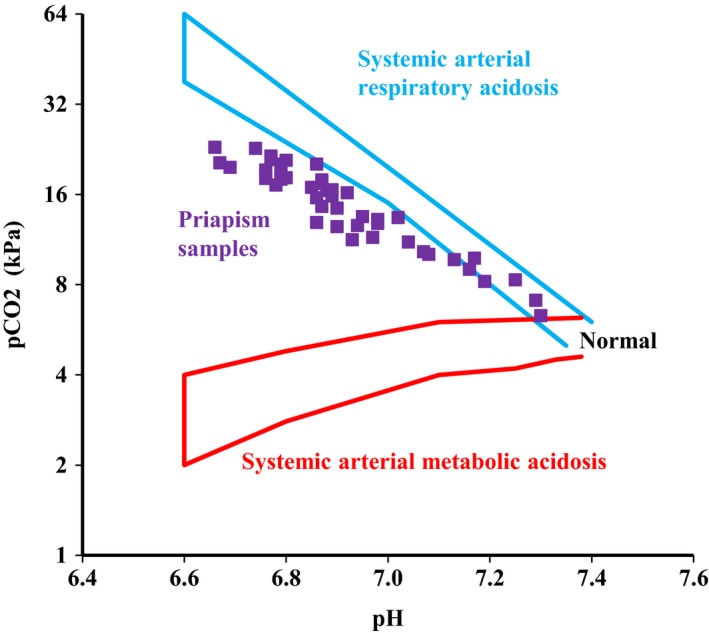
Plot of blood gas analyses of priapism samples on a nomogram for systemic arterial blood gasses. Adapted from (Ichihara et al. 1984).

We calculated the mean and standard deviation of the following variables: pH, pCO_2_, pO_2_, O_2_‐saturation, bicarbonate, base‐excess (BE), glucose, and lactate. We plotted the acid‐base status of each sample on an adapted acid‐base nomogram to identify the main acid‐base derangements (Fig. 1) (Spycher and Hauri 1986). We also determined relationships between pH and pCO_2_, pH and lactate, lactate and glucose, O_2_‐saturation and pCO_2_, and between O_2_‐saturation and glucose.

After obtaining the diagnostic blood sample, ischemic blood was aspirated with a 60 mL syringe through the same needle we used for the biochemical analysis. After the aspiration, 200 *μ*g of phenylephrine diluted by 1 mL of NaCl 0.9% was injected into the CC. If needed, this procedure was repeated several times (up to a maximum dosage of 1 mg of phenylephrine) until a stable flaccid state was achieved.

To compare the effect of in vitro addition of lactic acid (HLa) to blood with the in vivo measurements, we used anonymized spent arterial blood from routinely obtained blood gas samples and pooled them. Various doses of HLa (100 mmol/L) were added to arterial blood in a closed test tube to prevent CO_2_ loss. These samples were measured on the same analyzers that were used for the IP samples. In addition to pH, pCO_2_, bicarbonate, and lactate concentrations were also recorded.

This study was approved by the institutional review board of the UMCG (METc 2015/357).

## Results

We analyzed a total of 54 samples obtained during 47 episodes of IP that occurred in 21 different patients with a mean ± SD age of 34 ± 14 years. The causes of the IP were postintracorporal injection of papaverine/phentolamine (14 episodes), sickle cell disease (three episodes), clozapine use (one episode), chlorprothixene use (one episode), alcohol/methylphenidate use (one patient with frequently recurring IP), and idiopathic (28 episodes). The mean duration of the episode of priapism was 9 ± 9.5 h, with a range of 3–48 h and a median of 5.5 (IQR 4–10) hours.

Mean pH, pCO_2_, bicarbonate, and BE in the first sample of an episode were 6.91 ± 0.16, 15.3 ± 4.4 kPa, 21.1 ± 2.4 mmol/L, and −17 ± 7.9 mEq/L, respectively (Table 1). These results would be compatible with a severe combined acidosis when interpreted as a systemic arterial sample in the acid‐base nomogram (Fig. 1) (Siggaard‐Andersen 1971).

**Table 1:** Biochemical results

Variable	*N*	Mean (SD)	Range	Arterial reference range
pH	47	6.91 (0.16)	6.66–7.30	7.35–7.45
pCO_2_, kPa mmHg	47	15.3 (4.4) 115 (33)	6.3–23 47–173	4.6–6.0 35–45
pO_2_, kPa mmHg	47	2.43 (2.04) 18 (15)	0.18–7.80 1.4–59	9.5–13.5 70–100
O_2_‐saturation, %	45	18 (24)	1–86	96–100
Bicarbonate, mmol/L	47	21 (2)	16–26	21–25
Base‐excess, mEq/L	46	−17 (7.7)	−34.9 to −0.8	−3–+3
Glucose, mmol/L mg/dL	42	1.1 (1.5) 20 (27)	0.0–5.1 0–92	4.0–6.5 72–117
Lactate, mmol/L	45	14.6 (4.8)	4.6–23.0	0.5–2.2
Potassium, mmol/L	44	5.1 (2.1)	3.3–13.0	3.5–4.5

Results from the aspirated blood from the corpora cavernosa during 47 episodes of ischemic priapism. Note the extreme values when compared to the arterial reference range. The pH, pO_2_, O_2_‐saturation, base‐excess, and glucose are very low while lactate and pCO_2_ are very high. Bicarbonate and potassium were close to normality.

Mean pO_2_, O_2_‐saturation, and lactate were 2.4 ± 2.0 kPa, 19 ± 24%, and 14.6 ± 4.8 mmol/L, respectively, reflecting severe hypoxia–anoxia with lactic acidosis.

**Figure 2:**
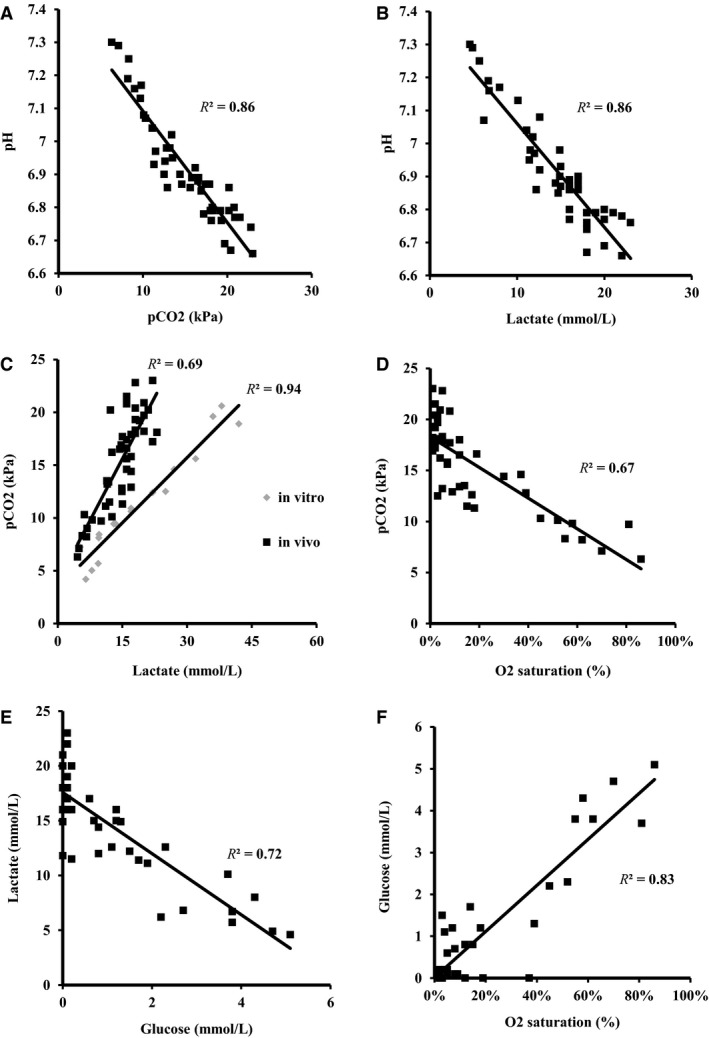
Various relations between metabolic parameters; (A) inverse linear relationship between pH and pCO_2_; (B) inverse linear relationship between pH and lactate; (C) comparison of in vitro and in vivo relationships between pCO_2_ and lactate. The slope in vivo is higher, indicating that there is residual oxidative metabolism with additional production of CO_2_; (D) inverse relationship between pCO_2_ and O_2_‐saturation; (E) inverse relationship between lactate concentration and glucose concentration indicating production of lactate with consumption of glucose; (F) relationship between glucose concentration and O_2_‐saturation indicating simultaneous glucose and oxygen consumption.

pH showed an inverse relationship with both pCO_2_ (*R* ^2^ = 0.86; *P* < 0.001) (Fig. 2A) and lactate (*R* ^2^ = 0.86; *P* < 0.001) (Fig. 2B). In both in vivo and in vitro experiments, there was strong positive relationship between lactate and pCO_2_ (Fig. 2C), with a steeper slope in vivo than in vitro.

Glucose levels were extremely low: 1.1 ± 1.5 mmol/L. O_2_‐saturation was negatively correlated with pCO_2_ (*R* ^2^ = 0.67; *P* < 0.001; Fig. 2D) and O_2_‐saturation was positively correlated with glucose (*R* ^2^ = 0.83; *P* < 0.001; Fig. 2F). Glucose and lactate were strongly inversely correlated (*R* ^2^ = 0.84; *P* < 0.001) with a slope of approximately ‐2.7 (Fig. 2E). The pH was positively related with BE (*R* ^2^=0.80; *P* < 0.001).

There was no correlation between the duration of priapism and metabolic variables (data not shown).

## Discussion

We found a remarkable combination of acidosis including both hypercapnia and hyperlactatemia, with glycopenia and hypoxia in the CC during IP (Table 1), indicating the exhaustion of all substrates required for ATP production. The various relationships between the metabolites are compatible with those expected for a closed system. This study on the intracorporal biochemistry during IP also underscores the important metabolic difference between *hypoxia* and *ischemia*. Whereas, the former relates to a shortage of oxygen, the latter can also involve depletion of fuel and accumulation of acid end products.

When the pH and pCO_2_ are plotted in an acid‐base nomogram, as is normally used for interpretation of systemic blood gas samples (Fig. 1), the priapism samples show a remarkable fixed relationship between pH and pCO_2_. Lactate levels were positively related with pCO_2_ levels in both priapism samples (in vivo) and during in vitro titration of normal blood with HLa in a closed system. (Fig. 2C). HLa is buffered by bicarbonate leading to a further rise of CO_2_ (as indicated by the equation below).

$${\text{HCO}}_{3}^{-}+H^{+}+{\text{La}}^{-}\leftarrow \to {\text{CO}}_{2}+H_{2}O+{\text{La}}^{-}$$

We believe that concomitant exhaustion of aerobic and anaerobic metabolism explains our observations. When the circulation in the CC stops, oxygen is consumed and pO_2_ and O_2_‐saturation drop with concomitant “aerobic” production of CO_2_. Glucose is also consumed in anaerobic metabolism leading to production of HLa.

The concomitant aerobic and anaerobic production of CO_2_ (i.e., resulting from the buffering of HLa by bicarbonate) explains the relatively stable bicarbonate levels, found across most of the pH range. Since these acid‐base disturbances occurred in a closed system, where CO_2_ could not escape, the actual metabolic character of the acid‐base disruption is completely different from an open system, such as a breathing patient (Ichihara et al. 1984). With increasing lactate levels, the PCO_2_ levels in vivo showed a more distinctive increase compared to those in vitro (Fig. 2C). This indicates that in vivo CO_2_ production is also the result of oxidative metabolism, whereas in vitro it was solely a consequence of the buffering effect of added HLa.

Hypoxia and acidosis cause diminished function in many organs and tissues, including the smooth muscle cells of the CC (Kim et al. 1996; Moon et al. 1999). The development of glycopenia in the CC during IP will further contribute to the impairment of smooth muscle contraction (Muneer et al. 2005). Since ATP is either generated by oxidative phosphorylation in the presence of oxygen or by glycolysis using glucose, severe local hypoxia and glycopenia result in a condition where ATP generation has become impossible. A metabolic endpoint is reached, which is rarely encountered in systemic blood samples of living individuals (Oldenbeuving et al. 2014).

The acidity of some samples in our study is noteworthy as they approached levels that are considered to be the lowest compatible with life (pH value of 6.6) (Bakker et al. 2013; Guyton and Hall 2016). For those cases where pH did not reach 6.6 we hypothesize that glucose availability, not acidosis, was an important limiting factor with regard of the recovery from IP.

To the best of our knowledge, there is only one previous study that describes the presence of glycopenia in CC blood samples obtained during prolonged priapism in six patients (Muneer et al. 2008). Glycopenia could potentially render the intracorporal administration of phenylephrine for restoring blood flow to the CC ineffective. In our study, the mean duration of IP was 9 h, whereas in the aforementioned study the mean duration of IP was 162 h.

As in IP, other models of regional ischemia have shown decreased tissue glucose levels with parallel increase in tissue lactate levels (Krejci et al. 2006). Some authors have suggested monitoring interstitial glucose levels to detect ischemia in closed systems such as compartment syndrome of the leg or venous and arterial occlusion after flap surgery (Sitzman et al. 2010; Doro et al. 2014). On the other hand in conditions involving impaired global circulation, acidosis often coincides with elevated glucose levels produced from hepatic glycogen stores, induced by adrenergic stress. In IP, obviously, no such compensatory mechanisms are available.

The observation of extreme glycopenia in the context of IP may also be relevant for clinical practice and management. The ATP‐deficient state will eventually lead to cell apoptosis of the spongious tissue cells in the CC, which renders the treatment with phenylephrine ineffective. Thus, it might be useful to dilute phenylephrine in glucose 5% (dextrose), instead of the usual NaCl 0.9%, to increase its effectiveness by recovering the glycopenic state and prevent cell apoptosis. In full erection, the volume of the CC varies from 36.9 to 87.5 mL (Wagner 1981). Thus the intracorporal administration of 1 mL of dextrose will increase the local glucose concentration by 3.5–7 mmol/L (60–125 mg/dL), that is, to physiological levels.

This study has a number of limitations. It is a retrospective analysis of a relatively large number of samples obtained from a smaller number of patients. We do not know how fast the described state of glycopenia, hypoxia, hyperlactatemia, and acidosis develops. A previous study in animals demonstrated that saturation drops to 65% after 180 min of penile clamp (Munarriz et al. 2003). Duration of priapism was determined from the onset, as reported by the patient, until the moment treatment was started. No relation was found between the duration of priapism and intracorporal blood chemistry.

In summary, careful biochemical examination of a closed compartment with a relatively high volume of blood revealed an extreme metabolic state. The severe combined respiratory and lactic acidosis with decreased or even absent oxygen and glucose levels emphasizes that all fuel reserves are metabolically exhausted. Whether the adjuvant treatment of glycopenia in IP with glucose offers a better outcome deserves further study.

## Conflict of Interest

None.
